# To what degree can variations in readmission rates be explained on the level of the hospital? a multilevel study using a large Dutch database.

**DOI:** 10.1186/s12913-018-3761-y

**Published:** 2018-12-27

**Authors:** Karin Hekkert, Rudolf B. Kool, Ester Rake, Sezgin Cihangir, Ine Borghans, Femke Atsma, Gert Westert

**Affiliations:** 1 Radboud University Medical Center, Radboud Institute for Health Sciences, IQ healthcare, Nijmegen, The Netherlands; 2 Dutch Health and Youth Care Inspectorate (IGJ), Utrecht, The Netherlands; 3 Dutch Hospital Data, Utrecht, The Netherlands

**Keywords:** Patient readmission, Healthcare quality indicator, Multilevel analysis

## Abstract

**Abstract:**

**Background:**

It is not clear which part of the variation in hospital readmissions can be attributed to the standard of care hospitals provide. This is in spite of their widespread use as an indicator of a lower quality of care. The aim of this study is to assess the variation in readmissions on the hospital level after adjusting for case-mix factors.

**Methods:**

We performed multilevel logistic regression analyses with a random intercept for the factor ‘hospital’ to estimate the variance on the hospital level after adjustment for case-mix variables. We used administrative data from 53 Dutch hospitals from 2010 to 2012 (58% of all Dutch hospitals; 2,577,053 admissions). We calculated models for the top ten diagnosis groups with the highest number of readmissions after an index admission for a surgical procedure. We calculated intraclass correlation coefficients (ICC) per diagnosis group in order to explore the variation in readmissions between hospitals. Furthermore, we determined C-statistics for the models with and without a random effect on the hospital level to determine the discriminative ability.

**Results:**

The ICCs on the hospital level ranged from 0.48 to 2.70% per diagnosis group. The C-statistics of the models with a random effect on the hospital level ranged from 0.58 to 0.65 for the different diagnosis groups. The C-statistics of the models that included the hospital level were higher compared to the models without this level.

**Conclusions:**

For some diagnosis groups, a small part of the explained variation in readmissions was found on the hospital level, after adjusting for case-mix variables. However, the C-statistics of the prediction models are moderate, so the discriminative ability is limited. Readmission indicators might be useful for identifying areas for improving quality within hospitals on the level of diagnosis or specialty.

**Electronic supplementary material:**

The online version of this article (10.1186/s12913-018-3761-y) contains supplementary material, which is available to authorized users.

## Background

Hospital readmissions are increasingly used as an indicator of the quality of care [1–5]. This is because premature discharge or substandard care during the initial hospitalisation has shown to increase the risk of readmission [6–9]. Furthermore, hospital readmissions are a burden to patients and are costly for the healthcare system [10]. The advantages of readmissions as an indicator are that they occur frequently, include a wide range of clinical diagnoses and most of the data needed to calculate readmission rates adjusted for their case-mix are already collected routinely [11]. Given the presumed negative relationship between readmissions and the quality of care, insight into the readmission rate might help hospitals to identify areas where the quality of their care can be improved [12].

Even though readmission indicators are already used worldwide, it is still not clear how much the indicator actually reveals about how far the hospital should be held accountable for readmissions. It is estimated that only around 30% of all readmissions are avoidable [10, 13–15]. The many different definitions used for readmissions also complicate their use as an indicator [2]. Also, the data collection methodology chosen (clinical reviewer method, administrative billing data method versus physician review of medical record) influences the differences in the rates of readmissions reported [16]. Despite these ambiguities, hospitals in the UK, US and Germany are already held accountable for high rates of readmissions for some diagnoses [17, 18]. In the UK penalties are imposed for high emergency readmission rates, while in the US this applies to the diagnosis groups: acute myocardial infarction, COPD, heart failure and pneumonia. In Germany hospitals only receive payments based on one diagnosis-related group (DRG) - that is to say only for the initial admission and not an eventual readmission. Readmissions are, therefore, paid for by the hospital [18].

It has now become necessary to understand the factors associated with readmissions because the readmission indicator is getting more and more popular and its consequences are increasingly far-reaching. A fundamental step is to understand to what extent hospitals themselves can influence the risk of readmission. Hospitals differ in readmission rates, but it is not clear yet which part of these differences is determined by the hospital itself. One technique to quantify the variation in readmissions on the hospital level is through multilevel analysis. This analysis takes into account the hierarchical structure of the admission data. Admissions are clustered within hospitals. Therefore, each observation is not independent. To take this into account, a couple of studies applied multilevel analysis in which they added a hospital level. Most of these studies focused on specific groups of diagnoses [19–24], or on a specific population of elderly patients [25, 26]. These studies found an ICC on the hospital level of around 1 to 5%. More extensive research is needed to investigate if this also applies with regard to the diagnosis groups that account for most readmissions.

The aim of this study is to assess the variation in readmissions on the hospital level after adjusting for the relevant case-mix in the Netherlands.

## Methods

### Data

We used data from the National Medical Registration (Landelijke Medische Registratie, LMR), one of the major Dutch administrative databases. This database provides data from 87 out of the 91 general and university hospitals in the Netherlands and contains all hospital admissions. We looked only at clinical admissions and excluded day care, which concerns, for example, patients undergoing outpatient surgery. This is because day care contains mainly planned admissions which are expected to have little effect upon the quality of care. We then extracted patients from the LMR database who were resident in the Netherlands for the period of 2010 to 2012. Patients not living in the Netherlands were excluded as either their index admission or their readmission could have taken place in their country of residence, and therefore readmissions could be underestimated. Dutch Hospital Data, the national organisation that administers the data from all the hospitals, gave permission to use the data anonymously.

### Definition, timeframe and inclusion/exclusion criteria

We defined a readmission as a clinical admission to the same hospital, within 30 days of discharge, following the clinical index admission, which is the original hospital stay. Patient identifiers are specific to an individual hospital and therefore it is only possible to look at readmissions within the same hospital. We chose this time frame in accordance with the international literature [15, 27]. Readmissions occurring within this time frame are likely to reveal weaknesses in the quality of care during the index admission [6, 28, 29]. We used the index admission as the unit of analysis, because this reflects better the clinical course of care. This means that each readmission of the same patient is again an index admission for a subsequent readmission [30].

We did not take into account whether the readmission was related to the previous hospitalisation, because no reliable method exists yet to select readmissions related to the previous principal diagnosis [27, 31]. Therefore, using our definition, the readmissions were ‘all-cause readmissions’. Acute admissions, as well as admissions which are not acute, were taken into account.

Admissions were included with a discharge date from 1 January 2010 until 31 December 2012. Furthermore, readmissions in January 2013 were included if they followed within 30 days of an index admission which had a discharge date in 2012.

Hospitals offering just one particular specialised form of care, such as ophthalmic surgery, were excluded from the dataset because they are not comparable with the general and university hospitals. Subsequently, we excluded hospitals with inadequate data quality. We investigated the following criteria, which are the same as those used for the calculation of the Hospital Standardised Mortality Ratio (HSMR) in the Netherlands [32], in order to assess data quality. There should be: at least six consecutive months of data registration, not more than 2% vague diagnoses, at least 30% acute admissions and, at least 0.5 comorbidities, on average, per admission. We assessed these variables - diagnosis, urgency and comorbidities - because they are subject to variations in coding between different hospitals as is known from the calculation of the HSMR. These variables are also important in the calculation of readmissions. Acute admissions and admissions with multiple comorbidities have a higher risk of readmission [1, 11]. Hospitals that did not meet one or more criteria were excluded from the analyses. Additionally, hospitals that registered a new patient ID at every admission were excluded because no readmissions could be identified. We focused on index admissions with surgical procedures with the highest number of readmissions in our analysis. Therefore, we only included hospitals which had a surgical procedure registered in at least 10 % of the admissions. This was because not all hospitals register procedures in the LMR. We compared the characteristics of the dataset used for analysis, with the dataset of hospitals that were excluded because they do not register procedures, in order to assess the comparability of both datasets.

Based on previous literature, we excluded admissions in which the principal diagnosis was either cancer care, obstetrics or psychiatric care [33]. For these patients, a major part of the readmissions is considered as necessary care. Cancer care and psychiatric care require follow-up care that is intrinsically clinically complex and extensive and therefore the degree to which it can be said to be preventable is difficult to assess. Obstetric readmissions are difficult to identify because most hospital deliveries in the Netherlands take place in the outpatient clinic and are therefore not registered in the LMR.

**Fig. 1:**
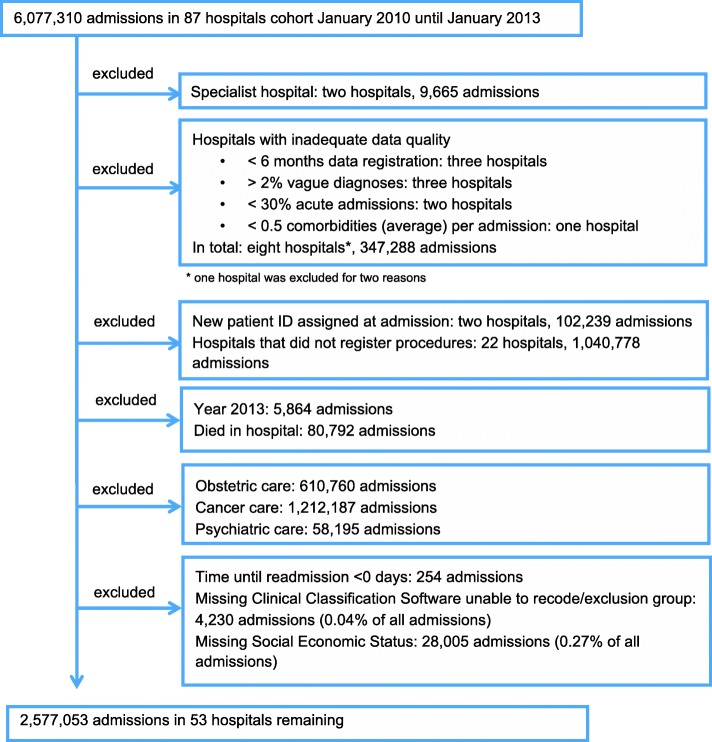
Flowchart admissions in dataset

Furthermore, patients who died during their index admission were excluded from the population at risk. Additionally, we excluded hospital admissions where their values were missing for one of the variables which were used in the logistic regression models. The number of hospitals and admissions excluded is shown in Fig. 1.

### Case-mix variables

We included the following predicting variables of the index admission in the model: age, gender, socioeconomic status (SES), urgency, year of discharge and comorbidities [34, 35]. The SES was derived from a table of postal codes from the Netherlands Institute for Social Research (SCP). These SES data were added to the database and provided five SES groups (lowest, below average, average, above average, highest).

The variable urgency (acute versus not acute) indicated whether care within 24 h was needed.

Comorbidities were assessed by the Charlson index [36], based on the secondary diagnoses for each admission. This index consists of 17 groups of comorbidities (Additional file 1), each being a separate case-mix variable. We assigned a 0 or 1 to each comorbidity group per admission to indicate the absence, or presence, of the comorbidity respectively. Secondary diagnoses, registered as a complication - coded with a ‘C’ added to the relevant secondary diagnosis - were not taken into account because they could be related to the quality of care in the hospital.

We included the year of discharge as a variable in order to take into account changes in the healthcare system every year due to new regulations and innovations. For example, when new coding rules apply and new financial incentives are created [37]. The date of discharge of the index admission determined to which year a record was assigned. We used the Clinical Classifications Software (CCS) to stratify for diagnosis [38]. This system consists of 259 diagnosis groups based on the International Classification of Diseases (ICD).

We did not take into account the length of stay, because it could be related to the quality of care within the hospital. Variation in the quality of the care, or its level of service, as with different waiting times for diagnostic tests or interventions, can affect the length of stay [39] and we did not want to correct for these differences.

### Analysis

#### Top ten diagnosis groups with large numbers of readmissions

We focused on index admissions with surgical procedures for the top ten diagnosis groups with the highest number of readmissions. This was because the literature indicates that unintended readmissions after surgical procedures are mainly the result of complications [40, 41]. The analysis was performed based on the diagnosis of the index admission. The readmissions, however, were all-cause readmissions. Procedures were registered in the LMR with CvV codes - a Dutch classification of procedures, used at the time of the research - and were classified as ‘surgical procedure’ or ‘no surgical procedure’. In order to compare hospitals that did register procedures and those that did not, we calculated the mean percentage of readmissions after an index admission with a ‘surgical procedure’ and the range of readmission rates across hospitals. We analysed Cohen’s d to calculate the effect size in order to analyse the relevance of the difference between the two datasets.

#### Variation on the hospital level

To assess the variation in readmission rates between hospitals, we performed multilevel logistic regression analyses, with a random effect on the hospital level. We did this for each of the top ten diagnosis groups. We also calculated these models without a random effect on the hospital level in order to assess the difference. In total, twenty models were calculated for each of the ten diagnosis groups, with and without a random effect for the hospital. We included in the models the case-mix factors: age, gender, SES, urgency, year of discharge and Charlson index (17 groups of comorbidities). This was in order to adjust for differences between hospitals in these factors. We scaled the variable, age, by calculating a z-score [42]. This appeared to be necessary to make the models fit, because this scale differed from the scales of the other case-mix variables. This standardisation puts the explanatory variables on an equal footing because it makes the scale of the variables irrelevant.

Case-mix variables with fewer than 50 admissions in a category were excluded from the models to prevent the standard errors of the regression coefficients becoming too large (a category is a combination of readmission yes/no and case-mix variable category) [43]. Comorbidities 9 and 17 (liver disease and severe liver disease) and 10 and 11 (diabetes and diabetes complications) were merged into one when there were fewer than 50 admissions where the comorbidity was present.

We then calculated the intraclass correlation coefficient (ICC) for each of the top ten diagnosis groups in order to assess the variation in readmissions between hospitals. This was achieved by using the method for calculating an ICC in the logistic multilevel models of Snijders and Bosker [44]. We calculated a C-statistic for the models including the hospitals and for the models not including this hospital effect. The difference between the two C-statistics was used as a measure for the contribution of the hospital to the variation in readmission rates.

We adjusted for case-mix in order to take into account the differences between hospitals regarding their patient population. We added only the significant predictors (*p* < 0.05) from the univariate analyses in the final models, because models which included all case-mix factors could not converge.

The data were analysed using R version 3.2.1. The package lme4 was used for the multilevel logistic regression and the package pROC was used to calculate the C-statistic.

## Results

A total of 6,077,310 admissions in 87 hospitals were present in the cohort from January 2010 until January 2013. In total three university and 31 general hospitals were excluded because of incomplete or incomparable data (The criteria are described in Fig. 1). The dataset used for further analyses consisted of 53 hospitals with, in total, 2,577,053 admissions of 1,784,709 patients. An overview of all the steps leading to exclusion, including the amount of admissions excluded in each step, is given in Fig. 1.

### Baseline characteristics

Table 1 gives the median, 5th and 95th percentile of the mean age, percentage women, percentage acute admissions, percentage acute readmissions and mean number of comorbidities per hospital. To calculate the comorbidities per hospital, we added up the comorbidities 1 to 17. It appears that there was some variation in age, gender, urgency of the admission, urgency of the readmission and comorbidities between the hospitals. The latter varied the most. The dataset of 22 hospitals that did not register procedures had comparable baseline characteristics, but the mean number of comorbidities was lower compared to the dataset of 53 hospitals. These differences were, however, not relevant. For all variables the Cohen’s *d* was around 0.

**Table 1:** Baseline characteristics, *N* = 53 hospitals

Variable	5th percentile	Median	95th percentile
admissions (N)	23,168	41,770	86,151
readmissions (N)	2236	4262	9182
% readmissions	8.68	10.16	11.89
mean age	45.33	51.28	54.84
% women	45.85	50.61	53.43
% acute^a^ admissions	41.73	55.61	67.58
% acute^a^ readmissions	49.20	68.87	78.12
mean number of comorbidities	0.06	0.22	0.44

^a^In the LMR an admission is registered ‘acute’ if care is needed within 24 h

### Variation on the hospital level

The top ten diagnosis groups was calculated using the index admissions with a surgical procedure that accounted for most readmissions. It appeared that the absolute number of readmissions after an index admission with a surgical procedure was highest in the diagnosis group ‘biliary tract disease’. The percentage of readmissions after an index admission with a surgical procedure was highest in the group ‘complications of surgical procedures or medical care’ (Table 2). See for the contribution of the case-mix variables of the models with hospital level Additional file 2.

**Table 2:** Admissions and readmissions per diagnosis group of the index admission, N = 53 hospitals

Diagnosis group^2^ (CCS^1^ code)	Admissions total (N)	Admissions with surgical procedure (N)	Readmissions after surgical procedure (N)	Readmissions and range(% of admissions with surgical procedure)
Biliary tract disease (149)	60.238	47.379	4.435	9.4 (4.7–13.3)
Osteoarthritis (203)	86.268	83.302	3.177	3.8 (2.4–7.4)
Complication of device; implant or graft (237)	40.625	25.374	2.929	11.5 (4.3–18.7)
Fracture of neck of femur (hip) (226)	31.672	29.136	2.242	7.7 (2.2–11.0)
Complications of surgical procedures or medical care (238)	36.835	13.265	1.972	14.9 (5.3–23.0)
Appendicitis and other appendiceal conditions (142)	27.247	24.546	1.751	7.1 (3.4–12.4)
Calculus of urinary tract (160)	20.344	11.300	1.412	12.5 (0.0–26.4)
Abdominal hernia (143)	26.286	23.647	1.421	6.0 (3.3–12.1)
Cardiac dysrhythmias (106)	92.360	15.129	1.239	8.2 (2.8–66.7)
Hyperplasia of prostate (164)	16.631	15.591	1.181	7.6 (3.2–17.5)

^1^*CCS* = Clinical Classifications Software [55]

^2^Diagnosis groups are sorted by number of readmissions after surgical procedure

Each of the index admissions can be also a readmission of a previous index admission. This is especially the case for the diagnosis groups ‘Complications of surgical procedures or medical care’ and ‘Complication of device; implant or graft’. Of these index admissions, 45% respectively 30% is also a readmission of a previous index admission. Therefore, a large number of the index admissions is also a readmission in these diagnosis groups. After adjusting for case-mix factors, the ICCs on the hospital level per diagnosis group ranged from 0.48 to 2.70% (Table 3). The C-statistics in the models with a random effect on the hospital level per diagnosis group varied between 0.58 and 0.65. The C-statistics in the models without a random effect on the hospital level varied between 0.52 and 0.64.

**Table 3:** ICCs hospital level and C-statistics of the models per diagnosis group of the index admission, N = 53 hospitals

Diagnosis group (CCS^1^ code)	ICC hospital (% and 95 CI)	C-statistic model with random effect hospital (95% CI)	C-statistic model without random effect hospital (95% CI)
Biliary tract disease (149)	0.48 (0.23–0.68)	0.651 (0.644–0.657)*	0.641 (0.635–0.648)
Osteoarthritis (203)	1.81 (1.26–2.41)	0.620 (0.611–0.630)*	0.597 (0.587–0.607)
Complication of device; implant or graft (237)	1.73 (1.22–2.15)	0.641 (0.634–0.649)*	0.625 (0.618–0.632)
Fracture of neck of femur (hip) (226)	2.33 (1.74–2.83)	0.617 (0.606–0.628)*	0.575 (0.564–0.586)
Complications of surgical procedures or medical care (238)	0.70 (0.45–1.15)	0.576 (0.568–0.584)*	0.556 (0.548–0.564)
Appendicitis and other appendiceal conditions (142)	1.45 (0.67–1.79)	0.583 (0.571–0.596)*	0.520 (0.506–0.533)
Calculus of urinary tract (160)	2.31 (1.57–2.64)	0.615 (0.604–0.625)*	0.574 (0.563–0.585)
Abdominal hernia (143)	1.36 (0.63–1.76)	0.648 (0.636–0.661)*	0.628 (0.615–0.641)
Cardiac dysrhythmias (106)	1.11 (0.65–1.18)	0.590 (0.585–0.595)*	0.568 (0.562–0.573)
Hyperplasia of prostate (164)	2.70 (1.37–3.86)	0.636 (0.620–0.651)*	0.592 (0.576–0.608)

^1^*CCS* = Clinical Classifications Software [55]

*significantly (*p* < 0.05) higher compared to the C-statistics of the models without a random effect on the hospital level

## Discussion

### Variation on the hospital level

the ‘biliary tract disease’ was the diagnosis group of the index admissions with a surgical procedure which was followed most often by a readmission in absolute terms. The percentage of readmissions varied slightly between hospitals. The range of readmission rates was greatest, but still small, for cardiac dysrhythmias. This means that there is some degree of variation between the hospitals in the number of readmissions for these cardiology diagnoses

The ICCs we found in this study (ranging from 0.48 to 2.70% for the diagnosis groups) are comparable with the study of Singh et al. who found an ICC on the hospital level of 0.84% in a Medicare population, which are mainly older patients [25], and a study of Jorgensen et al. who found an ICC of 1 to 2% in a cohort of colorectal cancer patients [20]. Burke et al. found a slightly higher ICC of 4.6% in a patient group hospitalised for ischaemic stroke [19]. ICCs on the hospital level for outcome measures are usually 1% or even 0.1% [20].

The C-statistics differed significantly for all diagnosis groups between the models with and without a random effect on the hospital level. The C-statistics of the models which included the hospital level were higher compared to the models which did not. The size of the difference varied between the different diagnosis groups. The higher C-statistics of the models which included the hospital level might indicate that part of the variation is explained by the hospital. This is especially the case in the diagnosis groups where the difference between the C-statistic of the model with hospital level and without hospital level is the largest: ‘appendicitis and other appendiceal conditions’, ‘hyperplasia of prostate’, ‘fracture of neck of femur (hip)’ and ‘calculus of urinary tract’.

The effect of the different patient characteristics varied between the models per diagnosis group (See Table 1, supplementary material).

We took the SES as a case-mix variable into account in our analysis as this is a relevant patient characteristic that influences the risk of a readmission [34, 35]. However, some studies showed that patients with a lower SES receive a lower quality of care [45, 46]. Therefore, it can be argued that SES should not be taken into account when calculating the indicator for use in practice as it is better not to adjust for this difference.

The C-statistics of the models with a random effect on the hospital level were modest ranging from 0.58 to 0.65, which is in accordance with the international literature [27, 34, 47]. The C-statistic was lower than in a comparable Belgian study which found a C-statistic of 0.73 [48]. These moderate C-statistics suggest that, given the predictors, the risk of readmission cannot be predicted accurately. Readmissions are probably influenced by other patient factors not available in administrative databases. This idea is supported by Barnett et al. who linked national survey data to that from Medicare claims. Of the 29 patient characteristics studied, 22 significantly predicted readmissions. Among these patient characteristics were social aspects, including marital status, employment status and having friends who are living nearby [49]. These variables were not available in our database.

### Implications for practice

Our study showed that, after adjusting for the relevant case-mix variables, a small part of the explained variation in readmission rates for some diagnosis groups exists on the hospital level. As the models for predicting readmissions show only moderate C-statistics, the discriminative ability is limited.

It is important to mention that the care provided in the immediate period after receiving hospital care can influence the number of readmissions. More specifically, this number depends on the destination of patients after discharge and the way the hospital arranges this care after discharge. Even if readmissions seem largely dependent on the patient’s health status or the quality of care after the patient’s discharge, hospitals can take responsibility for factors outside of their walls. For example, the hospital could improve the communication between the hospital and the community care physicians or by improving the discharge planning process. Several studies show that hospital strategies to reduce readmissions can be successful [50, 51]. For example, hospitals experienced significant reductions in unplanned readmission rates when they adopted the strategy of routinely discharging patients with a follow-up appointment already scheduled [50]. Therefore, the indicator could be used as a screening tool in the internal process of improving the quality of care [12] and also improving the aftercare and coordination in the healthcare chain. By identifying diagnosis groups and patient groups with a high risk of readmission, hospitals can take this into account in the planning of care for these patients and around their discharge.

### Strengths and limitations

Several studies calculated readmission rates without applying multilevel analysis [11, 14, 51]. However, it is necessary to use this technique, because of the hierarchical structure of the dataset. We also used the multilevel analysis to quantify the hospital contribution to the risk of readmission. Furthermore, several multilevel studies focused only on a specific diagnosis or patient group [19–26], while our study concerns ten different diagnosis groups in which readmissions are common after an index admission with a surgical procedure. The number of hospitals included in the database is another strength of this study. It contains admission data for more than half of the Dutch hospitals. Furthermore, data from three consecutive years were included for analysis. As the effect of the factor ‘year’ in the models is very small, we do not expect other results for more recent years.

Our study did not include the hospitals that did not register procedures. The characteristics of the 53 hospitals used for analysis were comparable to the characteristics of the 22 excluded hospitals which did not register procedures. However, the mean number of comorbidities was lower in these 22 hospitals. Therefore, the database used in this study might include relatively more severe patients. On the other hand, it could also indicate that these excluded hospitals did not register comorbidities completely.

Our study was limited to Dutch hospitals. It could be plausible that because of financial incentives in other countries, such as the UK and US, their readmission rates differ from those in our study. In addition, the number of admissions could be influenced by the way the immediate care after discharge from the hospital is arranged. In the Netherlands, general practice performs a strong gate keeping role and so many patients receive healthcare at home after discharge. Some systems, such as that in Belgium, which borders the Netherlands, are comparable. A Belgian study with similar methodology found a slightly lower acute readmission percentage compared to the Netherlands [48]. This might be because the registration of the urgency of admissions is not exactly the same. Furthermore, our results concerning the crude readmission rate and the ICCs are in line with the international literature. Therefore, we do not expect a different outcome when studying hospitals from other countries.

As this study is based on administrative data, it is important to minimise bias caused by differences in registration between the hospitals. Therefore, we excluded hospitals with inadequate data quality, so we expect that this did not affect our results.

However, our study was limited in its ability to track patients across hospitals because the database has no reliable information about transfers between different hospitals or readmissions to other hospitals. Nasir reported that 19% of the readmissions occurred in a different hospital and Halfon reported 17% [52, 53].

Another limitation concerned the lack of mortality data after discharge. A better estimate of the population at risk for readmissions could have been made with these data. Therefore, this can be included in further research by combining the medical database with the Dutch Municipal Personal Records Database (Gemeentelijke basisadministratie persoonsgegevens).

We could not exclude the intended readmissions from the indicator in this study. This is because it is difficult to identify intended readmissions - based on the available variables in the LMR - through the use of just one variable such as urgency. In the LMR an admission is registered ‘acute’ if care is needed within 24 h and therefore does not seem to reflect the difference between unintended and intended readmissions.

### Future research

It is necessary to exclude intended readmissions from the indicator in order to develop a readmission measure which reflects the quality of care. This is because they do not reflect poor quality of care [31]. Investigating patient records retrospectively can be seen as the gold standard for selecting unintended readmissions. However, this is a time-consuming procedure. Blunt et al. (2014) were able to classify preventable readmissions based on administrative data and to select readmissions for immediate reduction [15]. Furthermore, Goldfield et al. (2008) made an attempt to select potentially preventable readmissions (PPR) based on administrative data [54]. A recent study of Borzecki et al. examined whether the PPR algorithm distinguishes between good and bad quality of care on the individual case level in readmissions for pneumonia [55]. Based on administrative data, the PPR software matches the clinically-related index admission and readmission diagnoses which may indicate readmissions resulting from problems with the quality of care on admission or after discharge from hospital. They found no significant difference in the quality of care, as measured by processes of care received during the index admission and after discharge, between cases flagged as PPRs and those cases not flagged. This contrasted with their hypothesis. Therefore, reviewing medical records seems necessary in order to reveal the underlying causes of readmissions. This might be a crucial step in refining the readmission indicator. Furthermore, concerning case-mix adjustment, it is advisable to take into account the severity of the principal diagnosis in the prediction of readmissions. Finally, in order to understand potential differences in readmission rates between countries, a comparison between countries could be made.

## Conclusions

Our study showed that after adjusting for the relevant case-mix variables a small part of the explained variation in readmissions for some diagnosis groups can be found on the level of the hospital. However, the C-statistics of the prediction models are moderate, so the discriminative ability is limited. The modest contribution of the hospital level to a slightly better model indicates that there might be differences between the hospitals, especially for the diagnosis groups with the largest difference in C-statistic. A readmission indicator might be useful for identifying areas for improving the quality of care within hospitals on the level of diagnosis or medical specialty. Further research is needed to distinguish between intended and unintended readmissions.

## Additional files

Additional file 1: Charlson comorbidity groups with corresponding ICD9 codes (DOCX 17 kb)

Additional file 2: Contribution case-mix variables models with hospital level (DOCX 185 kb)
