# Supplementary material for: To what degree can variations in readmission rates be explained on the level of the hospital? a multilevel study using a large Dutch database
Source: BMC Health Serv Res. 2018 Dec 27;18:999. doi: 10.1186/s12913-018-3761-y (PMC6307249; doi:10.1186/s12913-018-3761-y)
Supplement: Supplementary file 1 — Charlson comorbidity groups with corresponding ICD9 codes (DOCX 17 kb) [file 12913_2018_3761_MOESM1_ESM.docx]

## Additional file 1. Charlson comorbidity groups with corresponding ICD9 codes [36]

| **Comorbidity** | **Charlson group** | **ICD9 codes** |
| --- | --- | --- |
| Comorbidity 1 | Acute myocardial infarction | 410, 412 |
| Comorbidity 2 | Congestive heart failure | 428 |
| Comorbidity 3 | Peripheral vascular disease | 441, 4439, 7854, V434 |
| Comorbidity 4 | Cerebral vascular accident | 430–438 |
| Comorbidity 5 | Dementia | 290 |
| Comorbidity 6 | Pulmonary disease | 490, 491, 492, 493, 494, 495, 496, 500, 501, 502, 503, 504, 505 |
| Comorbidity 7 | Connective tissue disorder | 7100, 7101, 7104, 7140, 7141, 7142, 71481, 5171, 725 |
| Comorbidity 8 | Peptic ulcer | 531, 532, 533, 534 |
| Comorbidity 9 | Liver disease | 5712, 5714, 5715, 5716 |
| Comorbidity 10 | Diabetes | 2500, 2501, 2502, 2503, 2507 |
| Comorbidity 11 | Diabetes complications | 2504, 2505, 2506 |
| Comorbidity 12 | Paraplegia | 342, 3441 |
| Comorbidity 13 | Renal disease | 582, 5830, 5831, 5832, 5836, 5837, 5834, 585, 586, 588 |
| Comorbidity 14 | Cancer | 14, 15, 16, 18, 170, 171, 172, 174, 175, 176, 179, 190, 191, 192, 193, 194, 1950, 1951, 1952, 1953, 1954, 1955, 1958, 200, 201, 202, 203, 204, 205, 206, 207, 208 |
| Comorbidity 15 | HIV | 042, 043, 044 |
| Comorbidity 16 | Metastatic cancer | 196, 197, 198, 1990, 1991 |
| Comorbidity 17 | Severe liver disease | 5722, 5723, 5724, 5728 |
