# Supplementary figures and images for: To what degree can variations in readmission rates be explained on the level of the hospital? a multilevel study using a large Dutch database
Source: BMC Health Serv Res. 2018 Dec 27;18:999. doi: 10.1186/s12913-018-3761-y (PMC6307249; doi:10.1186/s12913-018-3761-y)

**Additional file 2. Contribution case-mix variables models with hospital level**


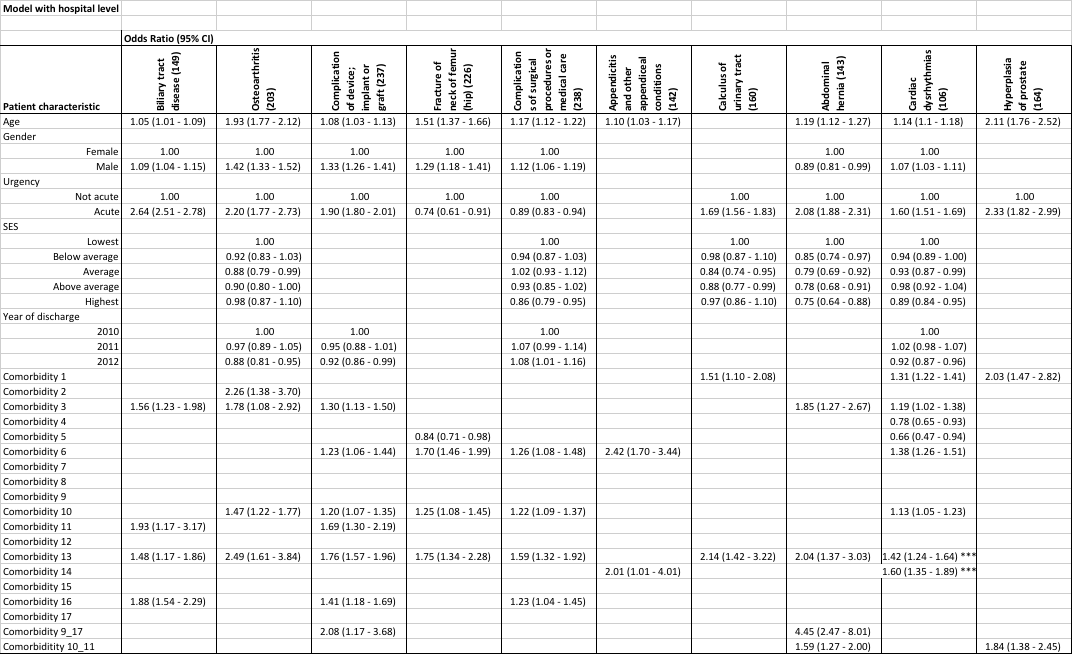

Supplement: Supplementary file 2 — Contribution case-mix variables models with hospital level (DOCX 185 kb) [file 12913_2018_3761_MOESM2_ESM.docx]
